# Comparison of the efficacy and safety of bupropion versus aripiprazole augmentation in adults with treatment-resistant depression: a nationwide cohort study in South Korea

**DOI:** 10.1192/j.eurpsy.2024.1815

**Published:** 2025-01-17

**Authors:** Dong Yun Lee, Rae Woong Park, Soo Min Jeon

**Affiliations:** 1 Department of Biomedical Informatics, Ajou University School of Medicine, Suwon, South Korea; 2 Bongdam Forest Mental Health Clinic, Hwaseong, Republic of Korea; 3 Department of Biomedical Sciences, Ajou University Graduate School of Medicine, Suwon, South Korea; 4 Jeju Research Institute of Pharmaceutical Science, College of Pharmacy, Jeju National University, Jeju, South Korea

**Keywords:** antidepressants, augmentation, depression, treatment-resistant depression

## Abstract

**Background:**

Treatment-resistant depression (TRD) affects 10–30% of patients with major depressive disorder, leading to increased comorbidities, higher mortality, and significant economic and social burdens. This study aimed to compare the efficacy and safety of bupropion and aripiprazole as augmentation therapies for TRD.

**Methods:**

This population-based, retrospective cohort study included adults aged ≥18 years with a diagnosis of depressive disorder who met the criteria for TRD. Data were collected from a nationwide claims database in South Korea. Patients prescribed bupropion were matched 1:1 with those prescribed aripiprazole. Subgroup analyses were performed according to age. An as-treated analysis was performed as the primary analysis, and an intention-to-treat analysis was performed to identify different risk windows. The primary outcome was depression-related hospitalization, and the secondary outcomes were first-time diagnoses of movement disorder and seizure.

**Results:**

A total of 5,619 patients (bupropion: *n* = 1,568; aripiprazole: *n* = 4,051) were included in this study. Bupropion was associated with lower risks of hospitalization (hazard ratio [HR]: 0.51; 95% confidence interval [CI] 0.29–0.86) and movement disorders (HR: 0.56; 95% CI 0.36–0.85) than aripiprazole. No significant difference in seizure risk (HR: 0.65; 95% CI 0.30–1.31) was observed between the two treatments. The subgroup analysis of participants aged ≥60 years revealed no significant differences in the three outcomes between the two medications.

**Conclusions:**

Bupropion augmentation is associated with a significantly lower risk of depression-related re-hospitalization and movement disorders in patients with TRD. Therefore, bupropion augmentation can be a comprehensive treatment strategy for TRD.

## Introduction

Major depressive disorder (MDD) is a prevalent mental disorder and a leading cause of disability worldwide [1]. Treatment-resistant depression (TRD) affects approximately 10–30% of patients with MDD [2–4]. TRD is an MDD that fails to achieve clinically significant improvement after two or more antidepressant treatment courses [5, 6]. TRD is associated with a significantly increased risk of psychiatric or physical comorbidities [7], higher mortality, and increased suicide rates [7–9], contributing to significant economic and social burdens [10, 11]. Therefore, optimizing treatment strategies for TRD is necessary for improving outcomes and providing patients with more effective personalized care.

The Sequenced Treatment Alternatives to Relive Depression (STAR*D) trial reported that bupropion was an effective augmenting agent for TRD [12, 13]. Additionally, several randomized controlled trials (RCTs) have shown that aripiprazole augmentation is superior to placebo for treating depression [14]. However, evidence on which of these two treatments offers a more comprehensive approach to managing TRD is limited. A previous systematic review and meta-analysis of RCTs reported that aripiprazole might provide a more comprehensive antidepressant regimen than bupropion for patients with depression [15]. However, this superiority was observed only in response rates, not remission rates; MADRS score changes, and adverse events. Furthermore, most trials included patients with MDD or TRD without a clear focus on TRD, making it difficult to establish strong evidence specifically for TRD. The OPTIMUM trial focused on patients with TRD who had failed more than two courses of antidepressant treatment and showed no significant differences in well-being scores and remission rates between aripiprazole and bupropion [16]. Notably, this study included patients aged ≥60 years, leaving younger populations underrepresented.

To the best of our knowledge, no real-world study has investigated the efficacy and safety of bupropion and aripiprazole as augmentation treatments for patients with TRD. Therefore, this study aimed to compare the efficacy and safety of bupropion and aripiprazole as augmentation therapies in a large nationwide population-based cohort of patients with TRD.

## Methods

### Study design and database

This was a retrospective observational cohort study using a nationwide claims database of Health Insurance Review and Assessment Services (HIRA) in the Republic of Korea from January 2018 to April 2022. HIRA employed an age- and sex-stratified sampling method to create a representative sample of 10 million individuals, accounting for 20% of South Korea’s population. This comprehensive HIRA database contains complete health information, including pseudonymized personal identifiers, demographic data, medical diagnoses, and data on procedures and medications listed in national reimbursement catalogs. The database has been standardized to align with the Observational Medical Outcomes Partnership Common Data Model version 5.3.1. A more detailed description of the database used in this study can be found in a previous report [17]. This study was conducted in accordance with local laws and regulations and approved by local ethics committees (Ajou University Medical Center Institutional Review Board: AJOUIRB-EX-2023-552). This study was reported following the STROBE guidelines for cohort studies.

### Study population and exposure

The code lists are detailed in Supplementary Method 1. The study included adults aged ≥18 years with depressive disorder diagnoses who were prescribed bupropion or aripiprazole. The index date was defined as the date of the first exposure to the target drugs (bupropion and aripiprazole). Patients were divided into two groups: the bupropion and aripiprazole groups. Patients enrolled in the database for <1 year before the first date of the target drug prescription were excluded to ensure minimal validity for the initial diagnosis and baseline covariates. Furthermore, all patients had to meet the criteria for TRD. A standardized definition of TRD that reliably predicts clinical decision-making and health outcomes has not yet been established [18]. According to the secondary analysis of the STAR*D naturalistic trial, TRD was defined as a lack of success in two antidepressant treatment attempts at sufficient doses and durations [19]. When observational databases lack information on patients’ responses to treatments, failure is inferred when a new antidepressant is prescribed [20]. Therefore, in this study, TRD was defined as a history of using three or more different types of antidepressants prior to the index date. The number of different types of antidepressants was defined as the number of antidepressants at the ingredient level. To ensure that bupropion and aripiprazole could be used as an augmentation agent, only patients who were taking at least one antidepressant on the index date were selected. Additional criteria were added by referring to recent comparative studies on the use of aripiprazole and bupropion in TRD [21]. Specifically, patients with a history of other psychiatric disorders that could affect treatment outcomes, such as bipolar disorder, depression with psychotic features, schizophrenia or psychotic spectrum disorder, moderate-to-severe alcohol or substance (nontobacco) use disorder, delirium, and dementia, were excluded. Furthermore, patients with a history of extrapyramidal and movement disorders (SNOMED-CT codes corresponding to G20–G26 of ICD-10) and seizures, which corresponded to contraindications or intolerances to the study medications, were excluded.

### Outcomes and follow-up

All outcomes were defined based on their diagnostic codes according to the SNOMED-CT classification (Supplementary Method 1). The primary outcome was depression-related hospitalization, which was defined as any hospitalization with a depression diagnosis but without prior hospitalization in the previous 2 weeks. The secondary outcomes were movement disorders and seizures. Antipsychotics such as aripiprazole are associated with neurological side effects, including movement disorders and seizures [22], and bupropion, among antidepressants, is particularly linked to these side effects [23]. Therefore, movement disorders and seizures were examined as safety outcomes. All study outcomes were limited to new-onset events, except for depression-related hospitalization. Furthermore, the results were validated through analysis using onychomycosis as a negative control outcome.

The patients were followed from the day after the index date until the earliest occurrence of one of the following: the final date of observed treatment (using an “as-treated” approach), their last recorded observation in the database, and the occurrence of an endpoint event or a censoring event. Treatments were considered ongoing if the patients received a new prescription within 30 days after the end date of their previous prescription. Treatments were considered discontinued if no additional prescriptions were received within 30 days following the last prescription, with the discontinuation date marked as 30 days after the final administration. Censoring events were defined as events in which patients were exposed to a comparator treatment. Censoring that occurred in one group was independent of the censoring of matched patients in the other group.

### Statistical analysis

Categorical variables were presented as frequencies and percentages. The baseline characteristics were identified within 12 months before the index date. The propensity score (PS) was calculated to adjust confounding bias between the two groups [24] and to estimate the empirical equipoise. The two groups were defined as comparable when >50% of the patients in each comparative pair had preference scores ranging from 0.25 to 0.75 [25]. Lasso logistic regression was used to estimate the PS using age group (in 5 years), sex, year of the index date, Charlson Comorbidity Index, and all coded information of the diagnosis and drug. Diagnosis and drug use were dichotomized. Patients with no code were considered to have no disease or prescription. The study groups were matched 1:1 based on the PS. A variable was defined as balanced if its absolute standardized mean difference (aSMD) < 0.25 [26]. The outcome incidence rates per 1,000 person-years were estimated. The Cox proportional hazards model was used to calculate hazard ratios (HRs) with 95% confidence intervals (CIs). Only treatment was included as a covariate in the Cox model if the covariates were balanced. If not, unbalanced covariates were corrected using double adjustment in the Cox model [27]. The cumulative incidence was derived, and between-group differences were compared using the Kaplan–Meier curve and Log-rank test. A p-value <0.05 was considered statistically significant. Additionally, subgroup analysis was performed for patients aged ≥60 years.

### Sensitivity analyses

Sensitivity analyses were performed across different analytical settings: PS adjustment methods and follow-up strategies. The PS adjustment was varied by applying maximum matching (1:n matching) or stratification into five strata. Additionally, the follow-up strategy was changed to an intention-to-treat (ITT) approach to estimate the effect of being assigned to a particular treatment regardless of adherence [18]. In the ITT strategy, patients were limited to those observed for 1 year and followed up until the study end date or the occurrence of the outcome. All analyses were performed using R version 4.1.0 and its open-source statistical packages [28].

## Results

### Cohort characteristics

**Figure 1.**
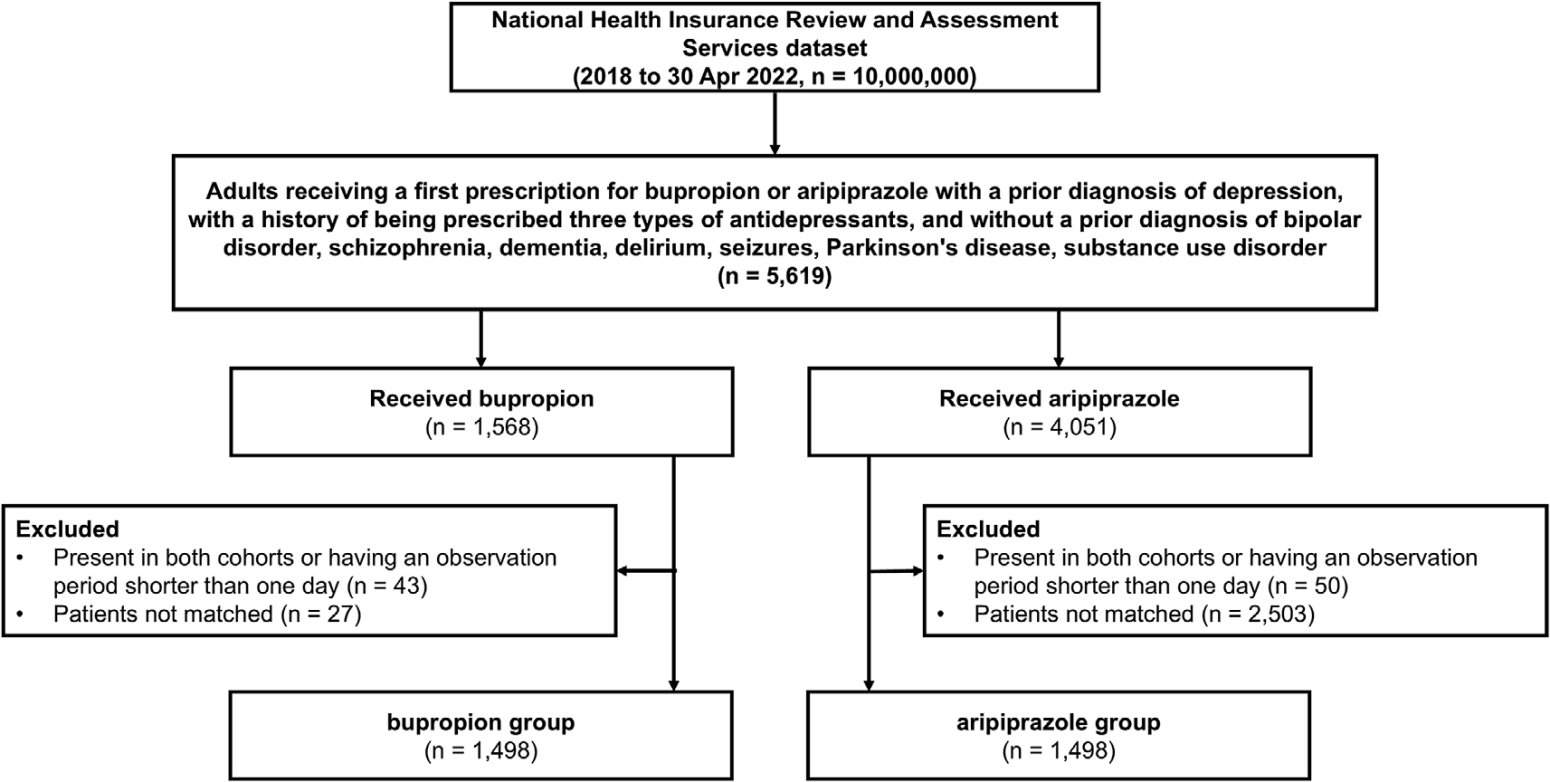
Flow diagram between the bupropion group and the aripiprazole group.

A total of 5,619 patients were included in the analysis. Among them, 1,568 (27.9%) patients were assigned to the bupropion group, and 4,051 (72.1%) were assigned to the aripiprazole group (Figure 1). After matching, the bupropion and aripiprazole groups included 1,498 patients. The median follow-up period was 35 (interquartile range, 14–182) days for the bupropion group and 57 (interquartile range, 18–241) days for the aripiprazole group. The study group pairs were comparable based on the empirical equipoise (Supplementary Figure 1).

Table 1 and Supplementary Table 1 show the baseline characteristics of the overall study population before and after PS matching. After PS matching, all baseline characteristics were balanced between 4,529 matched pairs for the bupropion and aripiprazole groups (all aSMD <0.25; Table 1 and Supplementary Figure 2). The proportions of males in the bupropion and aripiprazole groups were 33.4 and 32.8%, respectively. The ages of most patients in both groups ranged from 18 to 39 years (53.7 and 58.8%, respectively). SSRIs were the most frequently prescribed class of index antidepressant in both bupropion (72.5%) and aripiprazole group (83.4%). Mean dose of bupropion and aripiprazole prescribed was 144.5 mg (SD 69.3) and 2.4 mg (SD 5.5), respectively.

In the comparison of the subgroup by age (≥60 years), most baseline characteristics were balanced (most aSMD <0.25; Table 1 and Supplementary Figure 2). Some variables, such as the sex ratio, were not balanced even after matching, so double adjustment was applied. The proportions of males in both groups were 35.5 and 23.2%, respectively. In this subgroup, 147.6 mg (SD 68.6) and 2.6 mg (SD 2.8) were the mean doses of bupropion and aripiprazole prescribed at the index date, respectively.

**Table 1.** Comparisons of baseline characteristics, comorbidities, and concomitant drugs in adult patients with depression after propensity score matching

Characteristics	BPR (*n* = 1498), *n* (%)	ARP (*n* = 1498), *n* (%)	aSMD	BPR (≥60 years) (*n* = 259), *n* (%)	ARP (≥60 years) (*n* = 259), *n* (%)	aSMD
*Socio-demographics*						
Male	500 (33.4)	491 (32.8)	0.01	92 (35.5)	60 (23.2)	0.27
Female	998 (66.6)	1007 (66.2)	0.01	167 (64.5)	199 (76.8)	0.27
18–39 years	804 (53.7)	880 (58.8)	0.17	NA	NA	NA
40–59 years	437 (29.2)	364 (24.3)	0.19	NA	NA	NA
≥60 years	257 (17.1)	254 (16.9)	0.02	259 (100.0)	259 (100.0)	0.08
Race, Korean	1498 (100.0)	1498 (100.0)	0.00	259 (100.0)	259 (100.0)	0.00
*Comorbid mental health disorders*						
Anxiety disorder	864 (57.7)	855 (57.1)	0.01	163 (63.3)	162 (62.9)	0.01
Sleep disorder	714 (47.7)	687 (45.9)	0.04	144 (55.9)	133 (51.6)	0.09
Obsessive-compulsive disorder	37 (2.5)	53 (3.6)	0.04	4 (1.9)	5 (1.9)	0.01
Personality disorder	40 (2.7)	37 (2.5)	0.01	7 (2.7)	8 (3.1)	0.02
*Comorbid physical disorders*						
Hypertension	239 (16.0)	245 (16.4)	0.01	146 (56.4)	142 (54.8)	0.03
Diabetes mellitus	140 (9.4)	133 (8.9)	0.02	71 (27.4)	74 (28.6)	0.03
Ischemic heart disease	52 (3.5)	62 (4.2)	0.04	32 (12.4)	34 (13.1)	0.02
Chronic kidney disease	5 (0.4)	7 (0.5)	0.01	5 (1.9)	7 (2.7)	0.05
*Medication use*						
Anticholinergics	40 (2.7)	38 (2.6)	0.00	2 (0.7)	1 (0.4)	0.17
Antiepileptics	64 (4.3)	59 (4.0)	0.01	1 (0.4)	2 (0.7)	0.15
Anxiolytics	1351 (90.2)	1316 (87.9)	0.07	251 (96.9)	248 (95.8)	0.06
*Class of the index antidepressant*						
SSRI	1086 (72.5)	1249 (83.4)	0.26	176 (68.0)	200 (77.6)	0.21
SNRI	476 (31.8)	510 (34.1)	0.05	76 (29.7)	88 (34.1)	0.09
*BPR or ARP dose (mg)*						
Mean (SD)	144.5 (69.3)	2.4 (5.5)	NA	147.6 (68.6)	2.6 (2.8)	NA

BPR, bupropion; ARP, aripiprazole; aSMD, absolute standardized mean difference; SSRI, selective serotonin reuptake inhibitors; SNRI, serotonin and norepinephrine reuptake inhibitors.

### Outcome assessment

Regarding the primary outcome, a significant difference in hospitalization was observed between the bupropion and aripiprazole groups (HR: 0.51, 95% CI 0.29–0.86; 19 cases in the bupropion group vs. 45 in the aripiprazole group) (Table 2). Regarding the secondary outcomes, a significant difference in movement disorder was observed between the bupropion and aripiprazole groups (HR: 0.56, 95% CI 0.36–0.85; 32 cases in the bupropion group vs. 69 in the aripiprazole group). However, no significant difference in the risk of seizures was observed between the bupropion and aripiprazole groups. The negative control outcome did not differ significantly in any setting, including the sensitivity analyses (Table 2 and Supplementary Table 2). The subgroup analyses revealed no significant differences in the outcomes between the bupropion and aripiprazole groups (Table 2).

**Table 2.** Risk of outcome events between the bupropion and the aripiprazole group among total and subgroup

Outcomes	Total group^a^			Subgroup (≥60 years)		
	Incidence rate^b^			Incidence rate^b^		
	BPR (*n* = 1498)	ARP (*n* = 1498)	HR [95% CI]	BPR (*n* = 259)	ARP (*n* = 259)	HR [95% CI]
*Primary endpoints*						
Hospitalization^c^	48.01	87.78	0.51 [0.29–0.86]^d^	72.80	80.31	0.76 [0.23–2.31]
*Secondary endpoints*						
Movement disorder	82.09	136.80	0.56 [0.36–0.85]^d^	103.61	109.80	0.96 [0.35–2.47]
Seizure	27.70	42.60	0.65 [0.30–1.31]	35.56	48.99	0.46 [0.02–3.59]
*Negative control outcome*	68.61	51.57	1.11 [0.64–1.92]	119.98	82.14	1.14 [0.38–3.40]

Abbreviations: BPR, bupropion; ARP, aripiprazole; HR, hazard ratio; CI, confidence interval.

a Total group indicates all patients aged ≥18.

b Incidence rate was calculated as case per 1,000 person-years.

c Hospitalization indicates a hospitalization with the presence of a depression diagnosis; negative control outcome indicates onychomycosis. By using a negative control outcome, researchers can test whether an effect occurs that previous research suggests should not, allowing them to check for residual bias from unmeasured confounding.

d Statistically significant.

### Sensitivity analyses

**Figure 2.**
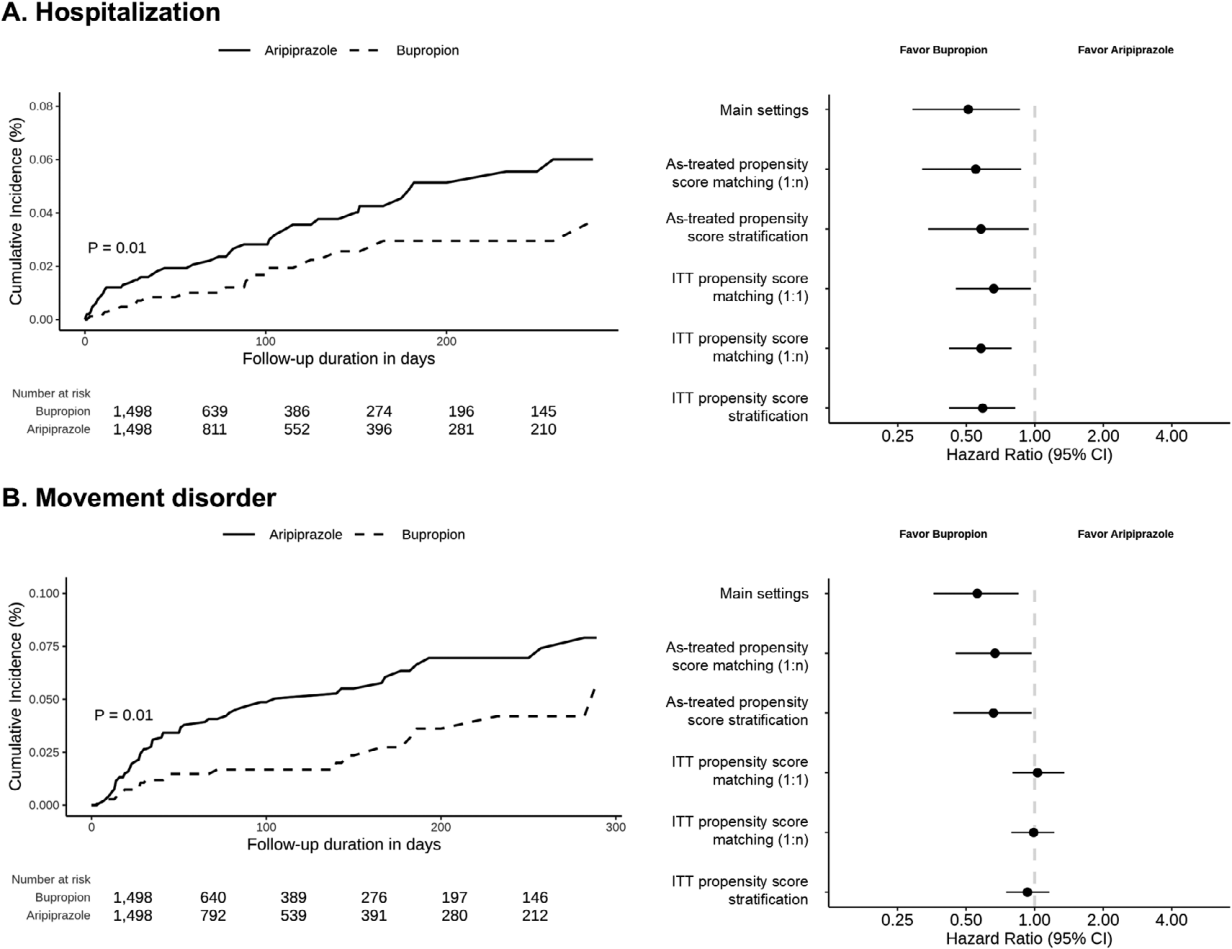
Comparison of hospitalization and movement disorder between the bupropion group and the aripiprazole group. (a) Kaplan–Meier plot and results of sensitivity analyses for hospitalization between the bupropion group and the aripiprazole group (b) Kaplan–Meier plot and results of sensitivity analyses for movement disorder between the bupropion group and the aripiprazole group. ITT, intention-to-treat. Hospitalization was defined as any hospitalization with a depression diagnosis but without prior hospitalization in the previous 2 weeks. Movement disorders were defined as the initial event occurring after medication use and include the concepts and subcategories of secondary parkinsonism, tremor, movement disorder, and dystonia as defined in SNOMED-CT.

Supplementary Tables 2 and 3 show the overall sensitivity analysis results. The risk of hospitalization (HR: 1:n matching, 0.55, 95% CI 0.32–0.87; stratification, 0.58, 95% CI 0.34–0.94; ITT with 1:1 matching, 0.66, 95% CI 0.45–0.96; ITT with 1:n matching, 0.58, 95% CI 0.42–0.79; ITT with stratification, 0.59, 95% CI 0.42–0.82) was consistently lower in the bupropion group than in the aripiprazole group (Supplementary Table 2). Additionally, the risk of movement disorders (HR: 1:n matching, 0.67, 95% CI 0.45–0.97; stratification, 0.66, 95% CI 0.44–0.97; ITT with 1:1 matching, 1.03, 95% CI 0.80–1.35; ITT with 1:n matching, 0.99, 95% CI 0.79–1.22; ITT with stratification, 0.93, 95% CI 0.75–1.16) was consistently lower in the bupropion group than in the aripiprazole group in the as-treated setting. However, no difference was observed in the ITT setting. Figure 2 shows the Kaplan–Meier curves of the main and sensitivity analyses for hospitalization and movement disorders. Regarding the seizure outcome, the results consistently showed no differences across the various sensitivity analysis settings. In the subgroup sensitivity analyses, the results consistently showed no differences across the various sensitivity analysis settings (Supplementary Table 3).

## Discussion

In this nationwide population-based cohort study, the efficacy and safety of bupropion and aripiprazole as augmentation treatments in patients with TRD were compared. Bupropion augmentation was associated with a lower risk of depression-related hospitalization than aripiprazole augmentation. Regarding safety, bupropion was associated with a lower risk of movement disorders, whereas no significant difference in seizure risk was observed between the two treatments. These results were consistently observed across various sensitivity analyses, which were performed using different analytical settings, including PS adjustments and the ITT approach.

This study showed that bupropion augmentation was superior for reducing the risk of depression-related hospitalization to aripiprazole augmentation. Given that hospitalization is influenced by a patient’s overall condition, such as symptom severity, comorbidities, and healthcare accessibility [29], the findings indicate that bupropion augmentation has a broader impact on stabilizing patient conditions and preventing severe relapses in patients with TRD.

However, this finding is inconsistent with that of previous studies [15, 16, 30–33]. This discrepancy may be due to several factors. First, this study specifically focused on patients with “pure” TRD, defined as failure to respond to two or more antidepressant treatments, whereas previous studies included broader populations comprising both TRD and those with general MDD. For instance, Cheon et al. included patients who failed only one antidepressant treatment strategy [30], which does not align with the widely accepted TRD definition. Therefore, our study might include patients with more severe symptoms and higher comorbidities, potentially contributing to the observed difference in outcomes.

Second, differences in study design may account for the contrasting findings. Although most previous studies were based on RCTs in controlled environments, this study used real-world data from a nationwide population-based cohort, which better reflects a more diverse patient population. Furthermore, whereas RCTs typically observed patients over short duration ranging from as short as 6 weeks to 3 months, our study included a longer observation period of up to 24 weeks in the as-treated analysis and up to three years in the ITT analysis. This extended observation period might have included long-term outcomes that may differ from those observed in RCTs. Additionally, whereas some RCTs involved large sample sizes of 1,500 participants and others were conducted with approximately 100 patients, our study included 3,000 matched patients, potentially leading to overall differences in patient characteristics. However, despite the large number of patients in our observational study, unmeasured confounders may not have been entirely excluded, highlighting the need for further consideration and additional research.

Third, there may be genetic differences in response to antidepressants. Existing RCTs are primarily conducted in the United States [15], representing the North American population. Given that previous studies have reported differences in antidepressant responses between Caucasians and Asians [34], these population-level differences might explain the variations observed in our results.

Fourth, differences in the prescription patterns of aripiprazole and bupropion in Korea may have an impact. According to Korea’s depression treatment algorithm, antidepressant monotherapy is recommended as the initial treatment strategy. In cases of severe symptoms, the use of antipsychotics is advised, with aripiprazole being the first-line antipsychotic. Conversely, bupropion is classified as a second-line antidepressant. Furthermore, until 2022, only psychiatrists were authorized to prescribe both antidepressants and antipsychotics in Korea. This restriction minimized worries about using antipsychotics, allowing aripiprazole to be commonly prescribed in line with treatment guidelines.

This study showed that aripiprazole augmentation was associated with a higher risk of movement disorders than bupropion augmentation. This finding is consistent with that of previous RCTs on patients with TRD [14, 31, 32]. Zisook et al. reported that aripiprazole augmentation was associated with more movement disorders, such as overall extrapyramidal effects and akathisia, than bupropion augmentation [32]. This difference in the risk of movement disorders may be due to distinct mechanisms. Based on receptor profiles, dopamine-blocking drugs, such as aripiprazole, reduce dopamine availability [35], which can lead to movement disorders, such as dystonia. In contrast, bupropion can increase dopamine levels and has been reported to modestly improve motor symptoms in patients with Parkinson’s disease [36]. Although some case reports have reported an association between the risk of movement disorders and bupropion, these cases are generally related to bupropion overdose or sudden discontinuation [37, 38]. In this study, the ITT analysis revealed that the increased risk of movement disorders for aripiprazole was not observed after discontinuation, indicating that this risk is limited to the active treatment period. These findings underscore the importance of closely monitoring movement disorders, specifically during aripiprazole treatment, and highlight the need for targeted prevention strategies.

Grand mal seizures have been reported to be a side effect of bupropion [39]. However, at the maximum daily dose of 450 mg of bupropion, the risk of seizures is 0.35–0.44%, similar to that of selective serotonin reuptake inhibitors [40]. Additionally, actual bupropion-related seizures are often due to overdose and tend to occur only in more susceptible individuals than in everyone [41]. In this study, the incidence rate of bupropion-related seizures was also relatively low. Furthermore, no statistically significant differences in the risk of seizures were observed between bupropion and aripiprazole, which has a relatively lower risk of seizures [42]. These findings indicate that although the risk of seizures associated with bupropion is well documented, it may offer similar safety in terms of the risk of seizures, thereby allowing for more flexibility in treatment selection based on individual patient needs.

In this study, subgroup analysis was performed on individuals aged ≥60 years. Pharmacological interventions are the most widely used treatments for late-life depression. However, special care is required when prescribing antidepressants to older people because they are more susceptible to drug-induced adverse events than younger adults [43]. This increased susceptibility may be due to physiological aging effects, such as diminished glomerular filtration, receptor density and activity changes, reduced liver size and hepatic blood flow, and decreased cardiac output. Considering these factors, a subgroup analysis was performed. The results showed that bupropion tended to be associated with a lower risk of hospitalization and movement disorders, although this was not statistically significant. Regarding seizures, no difference was observed between the two medications, which was consistent with the findings in the overall group. This tendency may not have reached statistical significance because of the insufficient number of patients in the subgroup analysis. Alternatively, the specific characteristics of age-related changes in older adults may have reduced the effects of the drugs, eliminating the actual differences between the medications [44]. Therefore, further verification with larger datasets is needed.

This study has some limitations. First, as this study was based on administrative claims data, we could not rule out the risk of under- or over-diagnosis, nor did we have information on the severity and symptoms of the patients. Additionally, the claims data did not provide information on treatment response and adherence, which could have influenced treatment outcomes. In this study we identified patients with TRD using proxy measures, which may not fully reflect actual treatment response. For instance, the European Group for the Study of Resistant Depression defined TRD as failure to response to two or more adequate trials of antidepressants from different classes, using specific numerical thresholds such as less than a 50% reduction on the Hamilton Depression Rating Scale or the Montgomery-Åsberg Depression Rating Scale after 6–8 weeks of treatment [45]. Moreover, our definition did not account for the current depressive episode in defining TRD. Therefore, further validation of this definition and the consideration of improved definitions are needed in future research. Second, using depression-related hospitalization as a surrogate for treatment efficacy may not fully capture the overall treatment efficacy. Third, although we adjusted for several variables to mitigate potential bias, some residuals may still exist due to differences in baseline characteristics. Additionally, unmeasured confounders, such as socioeconomic status and familiar history, may have influenced the outcomes.

In conclusion, bupropion augmentation was associated with a significantly lower risk of depression-related hospitalization and movement disorders than aripiprazole augmentation in patients with TRD. These findings indicate that bupropion augmentation is a more comprehensive treatment strategy for TRD. Further large-scale multicenter studies are needed to thoroughly evaluate the efficacy and safety of aripiprazole and bupropion augmentation in this population.

## Supporting information

Lee et al. supplementary material Lee et al. supplementary material

## Data Availability

Data are available from the corresponding authors upon reasonable request and with permission of HIRA.

## Supplementary material

The supplementary material for this article can be found at http://doi.org/10.1192/j.eurpsy.2024.1815.
