# Supplementary material for: Comparison of the efficacy and safety of bupropion versus aripiprazole augmentation in adults with treatment-resistant depression: a nationwide cohort study in South Korea
Source: Eur Psychiatry. 2025 Jan 17;68(1):e22. doi: 10.1192/j.eurpsy.2024.1815 (PMC11823002; doi:10.1192/j.eurpsy.2024.1815)
Supplement: Lee et al. supplementary material [file S0924933824018157sup001.docx]

**Supplementary Method 1. Code list for definitions**

| **Variables** | **Vocabulary** | **OMOP Vocabulary Codes** |
| --- | --- | --- |
| Bupropion | RxNorm | 750982 |
| Aripiprazole | RxNorm | 757688 |
| Antidepressants | ATC | 21604686 (ANTIDEPRESSANTS) |
| Subcategories of  antidepressants | RxNorm | 713109 (amoxapine),  710062 (amitriptyline),  750982 (bupropion),  797617 (citalopram),  798834 (clomipramine),  717607 (desvenlafaxine),  738156 (doxepin),  715259 (duloxetine),  715939 (escitalopram),  755695 (fluoxetine),  778268 (imipramine),  725131 (mirtazapine)  714684 (nefazodone),  721724 (nortriptyline),  722031 (paroxetine),  739138 (sertraline),  703547 (trazodone),  19041910 (tianeptine),  743670 (venlafaxine),  44507700 (vortioxetine) |
| Depression | SNOMED | 440383 (Depressive disorder),  442306 (Adjustment disorder with depressed mood),  4175329 (Organic mood disorder of depressed type) |
| Schizophrenia spectrum and bipolar disorder | SNOMED | 435783 (Schizophrenia),  4286201 (Schizoaffective disorder),  4335169 (Acute transient psychotic disorder),  35207135 (Shared psychotic disorder),  37117049 (Substance induced psychotic disorder),  434010 (Schizotypal personality disorder),  432590 (Delusional disorder),  436665 (Bipolar disorder) |
| Dementia | SNOMED | 4182210 (Dementia) |
| Delirium | SNOMED | 373995 (Delirium) |
| Seizure | SNOMED | 377091 (Seizure),  380378 (Epilepsy) |
| Extrapyramidal and movement disorders | SNOMED | 381270 (Parkinson’s disease),  4140090 (Parkinsonism),  374013 (Secondary parkinsonism),  378144 (Disorder of basal ganglia),  443782 (Tremor),  372604 (Movement disorder),  375800 (Dystonia) |
| Substance use disorder | SNOMED | 4319165 (Therapeutic drug dependence),  4217840 (Substance misuse behavior),  4279309 (Substance abuse),  443274 (Psychostimulant dependence),  4004672 (Psychoactive substance use disorder),  4219382 (Persistent substance misuse),  37116660 (Marijuana user),  440787 (Drug dependence in mother complicating pregnancy, childbirth AND/OR peurperium),  440069 (Drug dependence),  37116661 (Cocaine user) |
| Movement disorders | SNOMED | 374013 (Secondary parkinsonism),  443782 (Tremor),  372604 (Movement disorder),  375800 (Dystonia) |


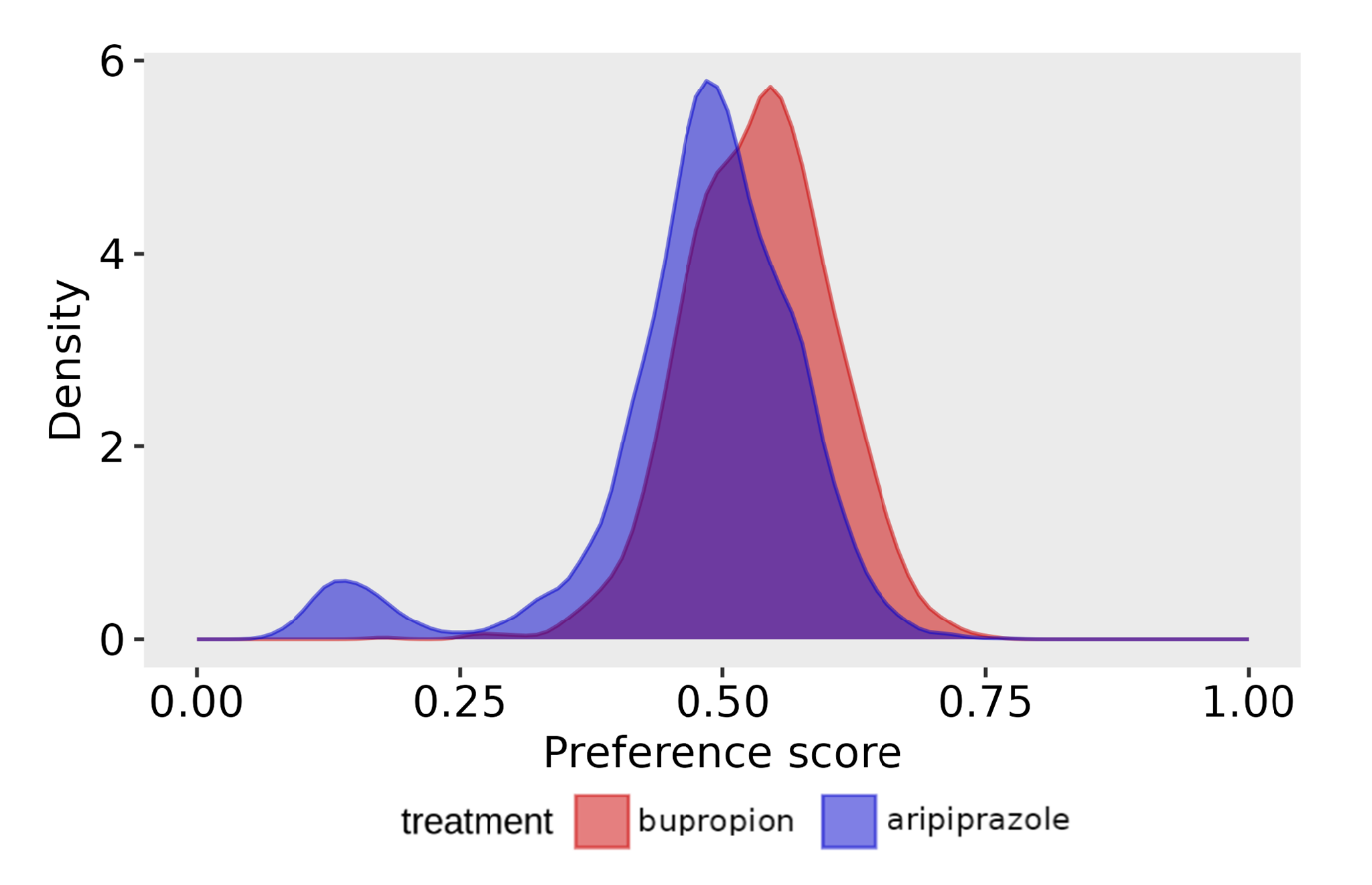


**Supplementary Figure 1. Empirical equipoise between the bupropion and aripiprazole groups**

**Supplementary Table 1. Comparisons of baseline characteristics, comorbidities, and concomitant drugs in adult patients with depression before propensity score matching**

| **Characteristics** | **BPR (n=1525), n (%)** | **ARP (n=4001), n (%)** | **aSMD** | **BPR (≥60 years) (n=259), n (%)** | **ARP (≥60 years) (n=690), n (%)** | **aSMD** |
| --- | --- | --- | --- | --- | --- | --- |
| **Socio-demographics** | | | | | | |
| Male | 514 (33.7) | 1240 (31.0) | 0.06 | 92 (35.5) | 180 (26.1) | 0.21 |
| Female | 1011 (66.3) | 2761 (69.0) | 0.06 | 167 (64.5) | 510 (73.9) | 0.21 |
| 18–39 years | 825 (54.1) | 2268 (56.7) | 0.10 | NA | NA | NA |
| 40–59 years | 442 (29.0) | 1044 (26.1) | 0.12 | NA | NA | NA |
| ≥ 60 years | 258 (16.9) | 689 (17.2) | 0.04 | 259 (100.0) | 690 (100.0) | 0.00 |
| Race, Korean | 1525 (100.0) | 4001 (100.0) | 0.00 | 259 (100.0) | 690 (100.0) | 0.00 |
| **Comorbid mental health disorders** | | | | | | |
| Anxiety disorder | 880 (57.7) | 2372 (59.3) | 0.03 | 163 (63.3) | 447 (64.8) | 0.03 |
| Sleep disorder | 727 (47.7) | 1912 (47.8) | 0.00 | 144 (55.9) | 372 (54.0) | 0.04 |
| Obsessive-compulsive disorder | 37 (2.4) | 128 (3.2) | 0.05 | 4 (1.9) | 9 (1.3) | 0.10 |
| Personality disorder | 41 (2.7) | 130 (3.2) | 0.03 | 7 (2.7) | 17 (2.5) | 0.02 |
| **Comorbid physical disorders** | | | | | | |
| Hypertension | 242 (15.9) | 656 (16.4) | 0.01 | 146 (56.4) | 397 (57.6) | 0.02 |
| Diabetes mellitus | 145 (9.5) | 356 (8.9) | 0.02 | 71 (27.4) | 195 (28.3) | 0.02 |
| Ischemic heart disease | 56 (3.7) | 172 (4.3) | 0.03 | 32 (12.4) | 96 (13.9) | 0.04 |
| Chronic kidney disease | 6 (0.4) | 16 (0.4) | 0.01 | 5 (1.9) | 15 (2.2) | 0.02 |
| **Medication use** | | | | | | |
| Anticholinergics | 39 (2.6) | 116 (2.9) | 0.02 | 2 (0.7) | 3 (0.4) | 0.17 |
| Antiepileptics | 64 (4.2) | 308 (7.7) | 0.15 | 1 (0.4) | 5 (0.8) | 0.17 |
| Anxiolytics | 1372 (90.0) | 3677 (91.9) | 0.06 | 251 (96.9) | 659 (95.5) | 0.07 |
| BPR: bupropion; ARP: aripiprazole; aSMD: absolute standardized mean difference. | | | | | | |


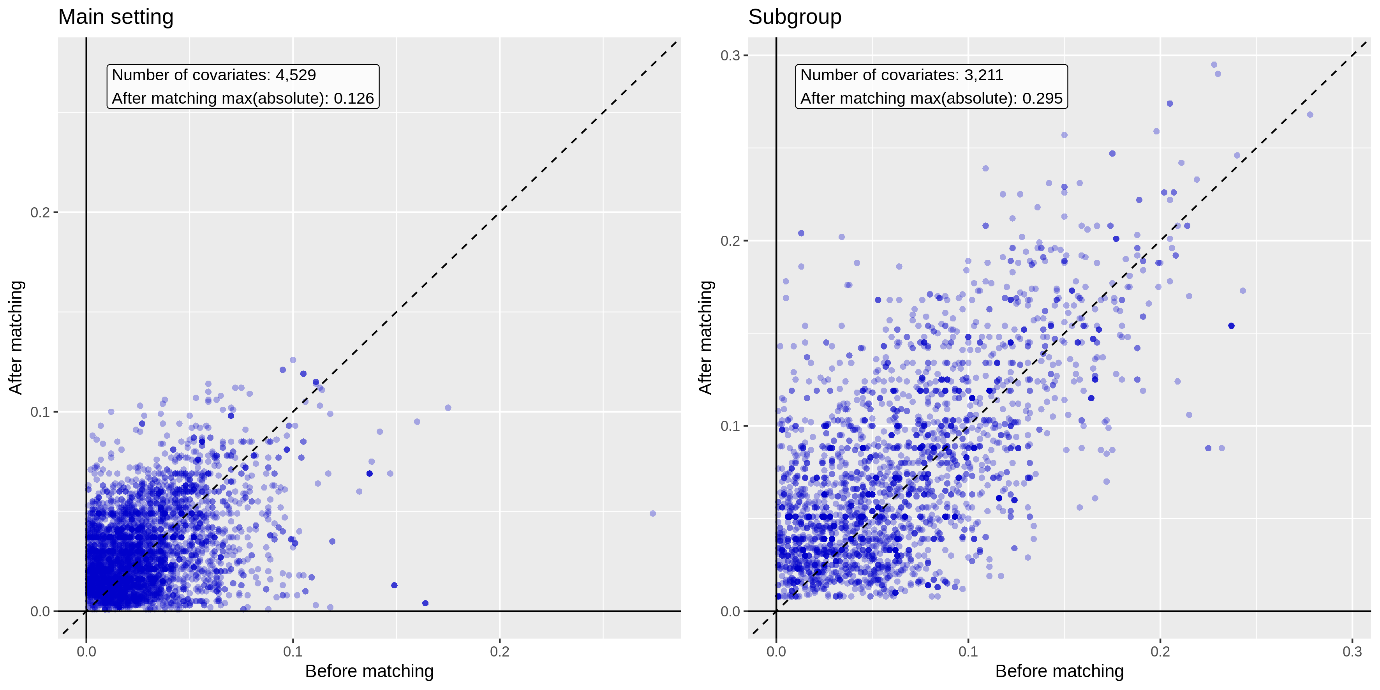


**Supplementary Figure 2. Scatter plots between before and after the propensity score adjustment between the bupropion and aripiprazole groups**

**Supplementary Table 2. Results of sensitivity analyses between the bupropion and aripiprazole groups**

| **Analysis description** | **Hospitalization** | **Movement disorder** | **Seizure** | **NC** |
| --- | --- | --- | --- | --- |
| **Main setting** | 0.51  [0.29–0.86] | 0.56  [0.36–0.85] | 0.65  [0.30–1.31] | 1.11  [0.64–1.92] |
| **AT PS matched (1: n)** | 0.55  [0.32–0.87] | 0.67  [0.45–0.97] | 0.71 [0.35–1.31] | 1.10  [0.69–1.71] |
| **AT PS stratified** | 0.58  [0.34–0.94] | 0.66  [0.44–0.97] | 0.79  [0.38–1.48] | 1.13  [0.70–1.80] |
| **ITT PS matched (1: 1)** | 0.66  [0.45–0.96] | 1.03  [0.80–1.35] | 1.33  [0.86–2.07] | 1.31  [0.94–1.83] |
| **ITT PS matched (1: n)** | 0.58  [0.42–0.79] | 0.99  [0.79–1.22] | 0.97  [0.69–1.36] | 1.15  [0.88–1.49] |
| **ITT PS stratified** | 0.59  [0.42–0.82] | 0.93  [0.75–1.16] | 1.03  [0.72–1.44] | 1.11  [0.84–1.45] |

**Supplementary Table 3. Results of sensitivity analyses between the bupropion and aripiprazole groups in the subgroup**

| **Analysis description** | **Hospitalization** | **Movement disorder** | **Seizure** | **NC** |
| --- | --- | --- | --- | --- |
| **Main setting** | 0.76  [0.23–2.31] | 0.96  [0.35–2.47] | 0.46  [0.02–3.59] | 1.14  [0.38–3.40] |
| **AT PS matched (1: n)** | 0.58  [0.20–1.39] | 1.00  [0.40–2.20] | 0.36 [0.02–1.90] | 0.92  [0.39–1.95] |
| **AT PS stratified** | 0.58  [0.20–1.39] | 1.00  [0.40–2.20] | 0.36  [0.02–1.90] | 0.92  [0.39–1.95] |
| **ITT PS matched (1: 1)** | 0.96  [0.51–1.77] | 0.88  [0.54–1.43] | 0.84  [0.34–2.04] | 1.14  [0.67–1.95] |
| **ITT PS matched (1: n)** | 0.87  [0.51–1.45] | 1.01  [0.65–1.52] | 0.99  [0.44–2.07] | 1.15  [0.73–1.76] |
| **ITT PS stratified** | 0.87  [0.51–1.45] | 1.01  [0.65–1.52] | 0.99  [0.44–2.07] | 1.15  [0.73–1.76] |
